# Distinctive incidence patterns of follicular lymphoma in Taiwan: Implications of ethnic differences

**DOI:** 10.1002/cam4.2028

**Published:** 2019-02-21

**Authors:** Shang‐Ju Wu, Yi‐Chu Chen, Wei‐Cheng Lo, Chun‐Ju Chiang, Chien‐Ting Lin, Shih‐Sung Chuang, Mei‐Shu Lai

**Affiliations:** ^1^ Division of Hematology, Department of Internal Medicine National Taiwan University Hospital Taipei Taiwan; ^2^ Graduate Institute of Epidemiology and Preventive Medicine, College of Public Health National Taiwan University Taipei Taiwan; ^3^ Taiwan Cancer Registry Center Taipei Taiwan; ^4^ Department of Pathology Chi‐Mei Medical Center Tainan Taiwan; ^5^ Taipei Medical University Taipei Taiwan; ^6^ National Taiwan University Taipei Taiwan

**Keywords:** age‐period‐cohort model, epidemiology, follicular lymphoma, incidence, survival, Taiwan

## Abstract

**Background:**

Follicular lymphoma (FL) is less prevalent in Asians, but detailed epidemiological analyses were not available. This study aimed to characterize the epidemiologic features of FL in Taiwan to explore the factors relevant to disease development and prognosis.

**Methods:**

We obtained epidemiological data for Taiwanese citizens during 1990‐2012 from Taiwan's National Cancer Registry Database, and the corresponding data for US Caucasians from the Surveillance, Epidemiology, and End Results Program. Changes in incidence rates were evaluated with age‐period‐cohort (APC) analyses. Patient outcomes were compared with 5‐year relative survival rates (RS) estimates.

**Results:**

Incidence rates of FL in Taiwan increased continuously during the study period (0.34 to 0.91 per 100 000 person‐year from 1993‐1997 to 2008‐2012 in men, and from 0.29 [1993‐1997] to 0.81 [2008‐2012] in women), while rates in the US remained stable in both sexes, ranging between 3.73 and 3.96 in men and between 3.24 and 3.55 in women. Estimates of average annual percentage changes in incidence were significantly positive in Taiwan, but not in US Caucasians. Notably, the APC analysis identified a strong birth‐cohort effect in Taiwan, corresponding to environmental alterations present during the study period. The estimated 5‐year RS rates in both populations showed steady improvement, but the RS in Taiwanese patients was consistently 10% lower than in US Caucasians.

**Conclusion:**

A distinct increasing trend of incidence with a strong birth‐cohort effect was identified in Taiwan, providing evidence of the association between environmental factors and disease development.

## 1 INTRODUCTION

Follicular lymphoma (FL), a non‐Hodgkin lymphoma (NHL) composed of follicular center B‐cells, is classified as either low‐grade (grades 1 and 2) or high‐grade (grades 3A and 3B) by the 2008 World Health Organization (WHO) classifications of lymphoid neoplasms. FL is common in the west, accounting for 20%‐35% of all NHL,^1^, ^2^, ^3^ while it is less prevalent in Asians and Blacks.^4^, ^5^ In the 1990s, the relative frequency of FL among all NHL in Taiwan was <10%.^2^ However, the relative frequency of FL in Taiwan has been increasing, and is now ranked as the second most common subtype, accounting for around 15% of all NHL.^6^, ^7^ Increased frequencies of FL are also observed in Japan.^8^ In addition, our prior study found a higher proportion of high‐grade tumors with a lower frequency of *t*(14;18)/*IGH‐BCL2* in Taiwan than in most Western countries.^9^, ^10^ These observations suggest that the biology of FL in Taiwan might be different and warrant the further exploration of the distinctions.

The age‐period‐cohort (APC) model, a classical epidemiological method used in analyzing temporal trends of incidence rates,^11^, ^12^, ^13^ is usually used to explore the potential effects of age, time‐period, and birth‐cohort on changes in incidence rates.^14^, ^15^ The age effect stands for physiological differences among different age groups in their susceptibility to a disease. The time‐period effect usually results from factors that equally influence all age groups during a given time‐period, including applications of new diagnostics or therapeutics, initiation of surveillance programs, or a short‐term dissemination of a carcinogen that may cause similar risk to everyone in a certain population. On the other hand, a birth‐cohort effect reflects factors that have different exposures in different birth‐cohorts, such as lifestyle factors that may be fixed early in life. Since lifestyle and environmental factors are hypothesized to be associated with the risk for developing lymphoid neoplasms,^11^, ^16^ it would be interesting to explore whether Westernized lifestyles and environment may contribute to the increasing frequency of FL in Taiwan.^3^, ^6^ The estimation of relative survival (RS) reflects the probability of cancer survival after adjustment for competing causes of death. RS is estimated as the ratio of observed survival to the survival rate that would have been expected if the cases had been subject only to age‐ and sex‐specific mortalities observed in the general population.^17^ RS reflects the probability of surviving the cancer of interest rather than the total survival probability. In this study, we applied the APC model to dissect the absolute incidence trends of FL and compared patient outcomes with their RS and explored the underlying factors contributing to differences in FL between Taiwan and the US Caucasians populations.

## 2 METHODS

### 2.1 Data source

Epidemiologic data on FL incidence rates in the Taiwanese population (ICD‐O M‐96903, 96913, 96953, 96983) during the period of 1993‐2012, as well as data on the survival outcomes of patients diagnosed between 1990 and 2009, were obtained from the National Taiwan Cancer Registry (TCR), a population‐based cancer registry founded in 1979 by the Ministry of Health and Welfare of Taiwan, which has been shown to be a high quality database.^18^, ^19^, ^20^, ^21^ More than 99% of FL cases in the database were histology confirmed between 1990 and 2012. The corresponding data in US Caucasians were obtained from the Surveillance, Epidemiology, and End Results (SEER) database from the US National Cancer Institute. The selection criteria were: SEER 9 and 18 registry, site, FL; race, non‐Hispanic White; and sex, male, and female.^22^, ^23^

### 2.2 Estimation of age‐standardized rates and average annual percentage change

Age‐standardized rates (ASR) according to diagnosis period were calculated by the direct method using the 2000 WHO world standard population.^24^ Trends in annual ASRs for persons between the ages of 30 and 79 years were analyzed using the annual per cent change and calculated using joinpoint regression analysis (Joinpoint Regression Program, Version 4.0.1, January 2013; Statistical Methodology and Applications Branch, Surveillance Research Program, National Cancer Institute, Bethesda, MD). The best fitting trend lines where the rates changed significantly were chosen by Monte Carlo permutation tests.^25^ The estimations were deemed statistically significant if the 95% confidence intervals did not include zero (*P* < 0.05).

### 2.3 The APC model

The model was fitted as a log‐linear model, which is a linear combination of age, time‐period, and birth‐cohort as follows:

$$\text{ln}\left(\lambda_{\mathrm{ijk}}\right)=\text{ln}\left(\mu_{\mathrm{ijk}}/{\text{n}}_{\mathrm{ijk}}\right)=\rho \;+\;\alpha_{i}+\beta_{j}+\gamma_{k}+e_{\mathrm{ijk}}$$

where *λ_ijk_*, μ*_ijk_*, and n*_ijk_* denote the incidence rate, the mean number of cases, and the number of individuals, respectively; *α_i_*, *β_j_,* and *γ_k_* represent the effects of the *i*th age group, the *j*th time‐period group, and the *k*th birth‐cohort group, respectively; *ρ* is the intercept term and *e_ijk_*is the random error term that follows a normal distribution with mean 0 and constant variance. Because of the linear dependence between age, time‐period, and birth‐cohort (age = time‐period − birth‐cohort), the estimate of the three factors cannot be identified. To overcome this problem, we used the methods proposed by Osmond and Gardner.^14^ The data from age groups younger than 30 were not included in the modeling because of the rarity of cases in these age groups. The period of 1998‐2002 and the birth‐cohort from 1941 to 1945 were used as reference groups. The relative risks (RR) for the time‐period and birth‐cohort were generated by the maximum likelihood method. Goodness‐of‐fit for each model was evaluated by comparing the deviance. A smaller deviance implies a better fit. The *F*test was used to test for significant differences in deviance between models. The analysis was conducted using apc.fit from the epi package in R (version 2.15.2; The R Foundation for Statistical Computing).

### 2.4 Relative survival estimation

Relative survival was defined as the observed survival among patients divided by the expected survival from the general population that is comparable with respect to the main factors affecting survival. Expected survival was derived from life tables stratified by age, sex, and calendar period using the Ederer II method. For survival analysis, the vital status of patients was ascertained using Taiwan's national death certificate database maintained by the Department of Statistics, Ministry of Health and Welfare. Patients were followed up for vital status until December 31, 2014. The survival time for each case was calculated as the time from the date of initial diagnosis to the date of death or follow‐up termination, whichever came first. Records were excluded if the date of birth or death was unknown. RS analyses were performed using SAS version 9.3 (SAS Institute, Cary, NC) and SEER*STAT (Surveillance Research Program, National Cancer Institute SEER*Stat software version 8.3.4, seer.cancer.gov/seerstat).

## 3 RESULTS

### 3.1 Increasing incidence of FL in Taiwan

**Figure 1:**
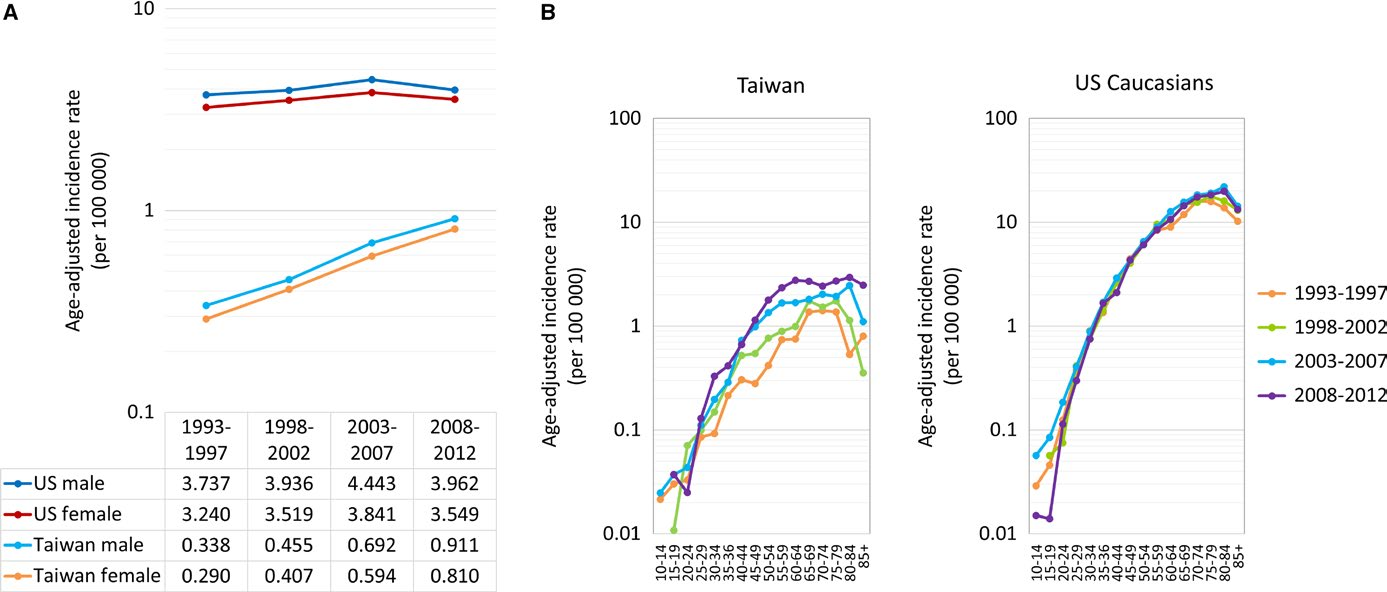
Average age‐adjusted incidence rates (A) and age‐specific incidence rates (B) of FL in Taiwan and among US Caucasians

Between 1993 and 2012, 2461 Taiwanese patients (1314 men and 1147 women) were diagnosed as FL, while 17 402 Caucasian patients in the US were diagnosed as FL during the same period (8581 men and 8821 women). The average age‐adjusted incidence of FL (per 100 000 people per year) in Taiwan increased from 0.34 (1993‐1997) to 0.91 (2008‐2012) in men and from 0.29 (1993‐1997) to 0.81 (2008‐2012) in women (Figure 1A). On the contrary, the incidence rates in US Caucasians remained steady in both sexes, ranging between 3.73 and 3.96 in men and between 3.24 and 3.55 in women. In every individual age group, the age‐specific incidence rate of FL in Taiwanese patients was lower than the corresponding rate in US Caucasians (Figure 1B). During the 20‐year study period, the incidence rates in Taiwanese patients peaked between 60‐70 years of age, whereas rates in US Caucasians peaked after 70 years of age, suggesting that Taiwanese FL patients tended to be younger than US Caucasian patients.

### 3.2 Increasing secular trend of FL incidence among all age groups in Taiwan

**Figure 2:**
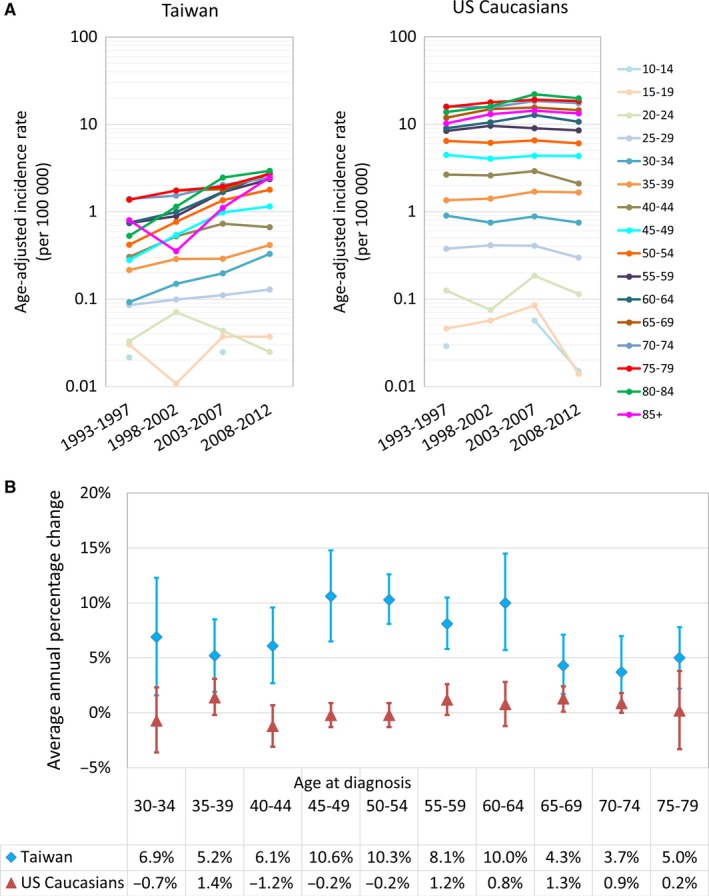
Secular trends in age‐specific incidence rates (A) and comparison of the AAPC in incidence rates (B) of FL in Taiwan and among US Caucasians

Figure 2A depicts the incidence trends for every age group, across different time‐periods. In both the US and Taiwanese adolescents and young adults, case numbers were sparse, and therefore, trends in these age groups were unstable for interpretation. In US Caucasians, the incidences in most age groups remained rather stable across the 4 study periods, whereas incidence rates in Taiwan increased consistently over time for every adult age group. Comparisons of the average annual percentage change (AAPC), which quantifies the time trends over the study periods, revealed that the estimated annual increase in incidence was significantly greater than zero in every Taiwanese age group, whereas the AAPC for US Caucasians was much lower and without a consistent pattern (Figure 2B). These are consistent with the observed increasing secular trends of incidence in Taiwan and with the steady secular trends of incidence observed in the US (Figure 2A).

### 3.3 Strong birth‐cohort effect underlying trends of FL incidence in Taiwan

**Figure 3:**
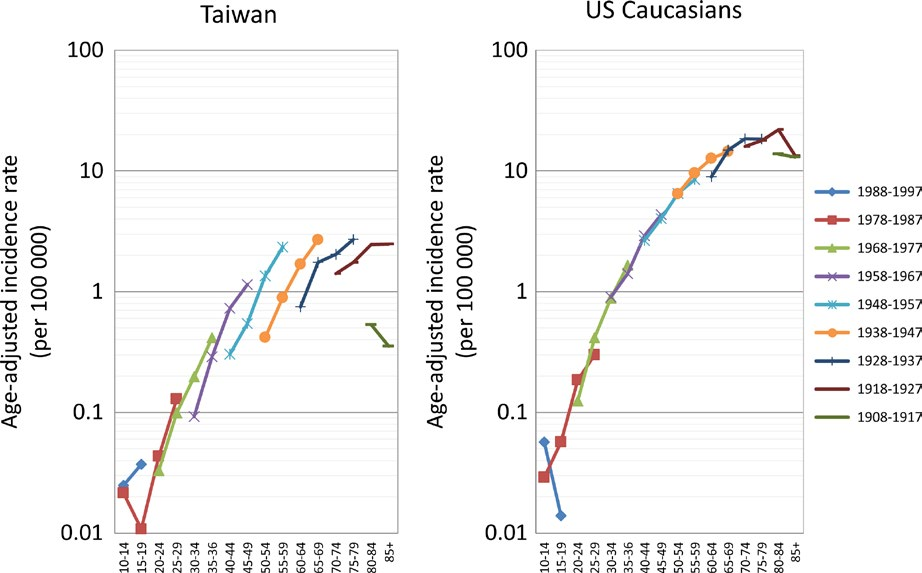
Age‐specific incidence rates of representative birth‐cohorts in Taiwan and among US Caucasians

**Figure 4:**
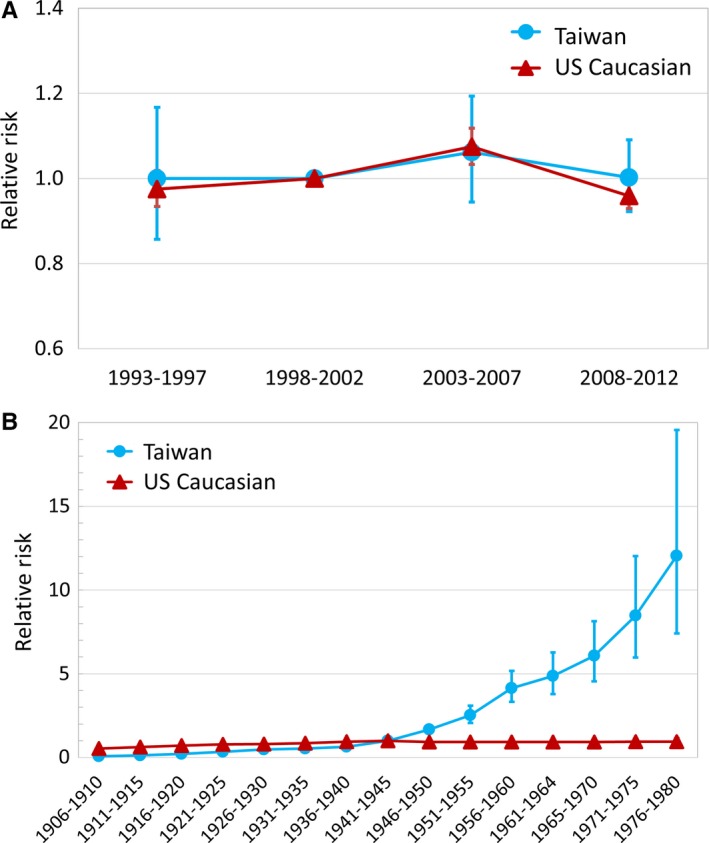
Comparison of period effects (A) and birth‐cohort effects (B) on the relative risk of FL between the Taiwanese and US Caucasians

Figure 3 demonstrates age‐specific incidences of FL in representative birth‐cohorts. For US Caucasians, little difference was seen in the incidences between earlier and later birth‐cohorts. In contrast, the incidences in later birth‐cohorts in Taiwan were higher than those of the earlier cohort in every given age group. Table 1 summarizes all possible models with APC effects. By comparing the deviance between adjacent lines (a lower *p*‐value indicates a better fit), it is possible to identify which model provides a better fit. In Taiwanese patients, the age‐cohort model, compared with the age‐alone or age‐drift models, reduced the residual deviances, whereas the influence of adding the time‐period factor on reducing deviance was less apparent, suggesting that a strong birth‐cohort effect underlies the incidence trends of FL in Taiwan. In US Caucasians, adding both factors reduced the deviance, but influences were much smaller because reductions were small. The comparisons of the RR derived from adopting the full APC model to reflect the individual effects of time‐period and birth‐cohort on FL in both cohorts are summarized in Figure 4. In Figure 4A, the absolute differences in RR among different time‐periods were small in both populations, a finding compatible with the statistics calculated in the goodness‐of‐fit test. On the other hand, the RR patterns across different birth‐cohorts were visibly different between Taiwanese patients and US Caucasians (Figure 4B). The cohort effect was not seen in US Caucasians, and the RR curve was nearly flat across different birth‐cohorts. In contrast, the RR curve in the Taiwanese population showed a continuous increase, suggesting the existence of a strong birth‐cohort effect underlying the epidemiology of FL in Taiwan.

**Table 1:** Comparing goodness‐of‐fit for different models

Model	Taiwan		US Caucasians	
	Residual deviance	Degrees of freedom	Residual deviance	Degrees of freedom
1. Age	361.39 (40)	40	167.351 (40)	40
2. Age‐drift	57.43 (39)	39	138.382 (39)	39
3. Age‐period	56.35 (37)	37	94.737 (37)	37
4. Age‐cohort	26.72 (33)	33	85.812 (33)	33
5. Age‐period‐cohort	25.10 (31)	31	51.139 (31)	31
	*P*‐value			
Model 2 vs model 1^a^	<0.001		<0.001	
Model 3 vs model 2^a^	0.5814		<0.001	
Model 4 vs model 2^a^	<0.001		<0.001	
Model 5 vs model 3^a^	<0.001		<0.001	
Model 5 vs model 4^a^	0.4460		<0.001	
Best‐fitting model	Model 4		Model 5	

a *P*‐values are derived from the *F*tests comparing the residual deviances between models.

### 3.4 Poorer survival outcomes for FL in Taiwan

**Figure 5:**
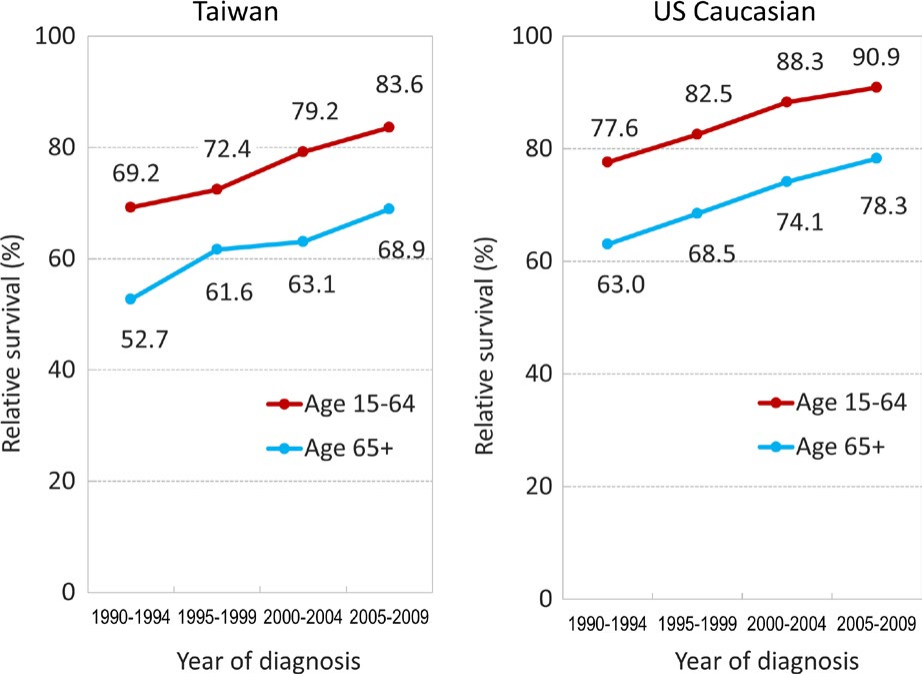
Five‐year relative survival estimates (%) of patients with FL during different time‐periods in Taiwan and among US Caucasians (1990‐2009)

In addition to the analyses of incidence rates, a parallel observation about FL patients' outcomes, as represented by 5‐year RS, was also analyzed (Figure 5). Among both populations, earlier time‐periods showed poor survival in both younger and older patient groups. Patient outcomes in both populations showed continuous improvement in the subsequent 20 years, and both age groups showed similarly parallel trends. However, the negative impacts from FL remained apparent, especially in older patients (52.7%‐68.9% for Taiwanese from 1990‐1994 to 2005‐2009, and 63.0‐78.3 for US Caucasians, respectively). In addition, a consistent difference in RS between Taiwanese and US Caucasian patients was seen across all 4 diagnosis periods: the RS estimates in Taiwanese patients were consistently 10% lower than estimates in US Caucasian patients of the same corresponding age group and during the same period.

## 4 DISCUSSION

Prior studies addressing the increase of FL in Asia have been based on the relatively ratios, that is, the ratio of FL among whole lymphoma cases.^6^, ^7^ In this study, we used incidence rate, an absolute parameter, which not only facilitates objective comparison between different populations but also makes statistical modeling more feasible and reliable. With this approach, this study confirmed the increasing trend of FL in Taiwan during the past 20 years, and contrasts with the stable incidence in US Caucasians. Furthermore, a stronger birth‐cohort effect was identified in the Taiwanese population, suggesting that changes in lifestyle and environmental exposures may play a role in the development of FL in this region. Finally, the outcome in Taiwanese FL patients was worse than in US Caucasians, indicating a larger unmet medical need in this region.

Interestingly, an earlier onset peak of FL at a relatively younger age group, almost 20 years younger, among Taiwanese was identified in our study (Figures 1B and 2A). Compared with earlier generations, the most drastic environmental changes in younger Taiwanese was the westernization in lifestyle, which took place after World War II, and accelerated after the 1960s when Taiwan started to industrialize.^11^, ^12^ Such westernization has been hypothesized to contribute to the increase in various cancers, such as chronic lymphocytic leukemia and female breast cancer.^11^, ^12^ Environmental factors that are common in westernized, industrialized environments have also been reported to be correlated with the risk of developing FL. For example, in the EpiLymph study, Cocco et al^27^ reported that occupational solvent exposure was associated with an increased risk of FL. Zhang et al also observed a positive association between hair dye exposure and risk of FL in the InterLymph program.^28^ Furthermore, Richardson et al reported that exposures to some chemical agents, such as arsenic and compounds, asbestos, diesel fuel, and nitrate, nitrite, or nitrosamine, were potential occupational risk factors for FL.^29^ High calorie intake was also reported as a risk factor for FL by Pan et al, and it was further supported by the fact that the prevalence of obesity in Taiwan increased sharply in recent surveys, from 11.8% in 1993‐1996 period to 22.0% in 2013‐2014 period, whereas the prevalence of obesity in the US did not change significantly.^30^, ^31^ Furthermore, there seemed to be an earlier onset peak at a relatively younger age groups in Taiwan (age 60‐64); this finding is in agreement with the significant cohort effects identified by APC modeling in Taiwan, suggesting that the environmental exposure appeared in earlier life might contribute more. Exposure to the westernization in 1960s in Taiwan earlier in life might result in a higher risk of developing FL after a latent period in the younger birth cohorts and lead to the observation that the increasing trend of FL was more prominent in the young population than in the elderly in Taiwan. Our findings are in line with those reported in prior studies and suggest that the birth‐cohort effect found in Taiwan may be a reflection of environmental influences from a westernized lifestyle and industrialized environment.

Despite the increasing trend, the overall incidence of FL remains lower in Taiwan than in US Caucasians. We speculated that this racial disparity is related to the genetic background. Recently we noted that the precursor condition for FL, that is, circulating lymphocytes carrying the *t*(14;18)‐*IGH/BCL2* translocation in healthy subjects, was much less prevalent in Taiwan than in the US. Interestingly, in Taiwan, the frequency of circulating lymphocytes with the *t*(14;18)‐*IGH/BCL2* translocation in the general population and the incidence of FL are both around one‐fourth of the US, suggesting that ethnic disparity begins in the very early stage of disease development.^33^ This finding is also compatible with the hypothesis that the molecular pathogenesis of Western and Asian FLs are distinct.^3^ It has also been reported by Herrinton et al that Asian immigrants in the US are at lower risk for FL, indicating the relevance of genetic background on the development of this disease.^34^ Furthermore, the risk for FL was found to be lower in first generation Chinese immigrants than in later generations, supporting the notion that differing environmental exposures might contribute to the differences in the incidence trends of FL among different generations.^34^ To further support this notion, we extracted corresponding data among American Indians, Alaska natives, and Asian‐Pacific Islanders from the SEER database. These populations are ethnically closer to the Taiwanese, but have been continuously exposed to a westernized lifestyle. We expected that their incidence rates of FL would be lower than US Caucasians but show a similar secular trend to the US pattern. As shown in Figure S2, the incidence rates of FL in these populations were in‐between the Taiwanese and US Caucasians, but the increasing trend was minimal, consistent with effects from genetic background and environmental factors, respectively. This finding is compatible with our hypothesis that the westernization of lifestyle may be associated with the cohort effect seen in Taiwan.

Some limitations of the incidence analyses should be addressed. It may be difficult to differentiate between a time‐sustained period effect, such as the modification of diagnostic criteria or improvement in access to medical care with better disease detection, and an actual cohort effect in the APC model. The REAL (Revised European‐American Classification of Lymphoid neoplasms)/WHO classification for lymphoid neoplasms was introduced in 2000/2001 and was revised in 2008. However, the basic diagnostic criteria for FL has remained essentially the same in these classification systems, and the same criteria are used in both countries. Besides, increasing access to medical care was occurring in both regions. Thus, the identified cohort effect is not supposed to be confounded by the aforementioned factors. Second, the completeness of individual registry systems may be of concern. However, underestimation of the incidence rates should not influence the long‐term trends, as long as the quality of the registry is stable. In this study, the incidence curves for both populations (Figure 1) were smooth without fluctuation, suggesting that the difference in incidence trends should not be significantly influenced by issues in ascertainment.

In addition to the incidence difference, outcomes of FL patients in Taiwan were distinctly poorer; apparent RS differences were seen among both younger and older patients. In fact, in other lymphoma subtypes, the outcomes are reported to be similarly poorer in Asian patients.^20^, ^21^, ^35^ In this study, RS, which reflects the probability of surviving the cancer of interest rather than the total survival probability, was applied for outcome comparison. This approach may eliminate the possible bias that the improvement in OS is in fact a reflection of the improvement of life expectancy of the whole population resulting from better general medical care, hygiene infrastructure, public health strategy, etc, that benefits the whole populations. We do not expect this to be a result of differences in the availability of definitive anti‐lymphoma therapies, because cytotoxic chemotherapy, rituximab, and hematopoietic stem cell transplantation were almost equally available in both countries during this study period. For example, rituximab, the frontline treatment for FL, was approved in the US in 2006; it was also during this same year that the treatment began to be reimbursed in Taiwan. Moreover, the rates of patients receiving systemic (chemotherapy and/or target) therapies derived from both databases demonstrated that, after the initiation of the National Health Insurance program in Taiwan in 1995, improved medical availability in Taiwan actually resulted in higher proportions of FL patients in Taiwan receiving systemic therapies after 1995 than in U.S. Caucasians (Figure S2). The underlying causes for the outcome differences thus do not result from the availability of therapeutic medicines. The other possible explanation is differences in the disease biology. The TCR does not collect detailed data for FL grades, information that is relevant to the prognosis, but a recent report showed that the proportion of high‐grade FL in Taiwan is larger than in Western cohorts,^10^ suggesting the possibility of differences in disease‐relevant biological factors. In addition, in this study, the outcome differences are similar in younger and older patients in different periods, suggesting the existence of some common, disease‐relevant biological factors in both younger and older patients that are contributing to the outcome differences. Finally, despite the similar availability of definitive anti‐lymphoma therapies, the differences in cancer supportive care may play an important role, especially in lymphoma with long disease courses. The disparity in patient prognosis seen in this study clearly demonstrates an unmet medical need and health disparity in FL in Taiwan that warrants further exploration. However, outcomes in either younger patients and older ones in both populations have been continuously improving. In addition to the improved outcomes observed in clinical trials,^36^, ^37^ modern advances in treatment for FL are noticeably improving outcomes of FL patients in the real world for FL patients in both younger and older age groups.

In conclusion, this study identified a distinct increasing trend of incidence with a significant birth‐cohort effect in Taiwan, providing evidence of the association between environmental factors and disease development. The improved RS rates imply that therapeutic advances are changing the clinical course of FL, but the sustained gap in RS between Taiwanese and US Caucasians suggest unmet medical needs in Taiwan.

## CONFLICT OF INTEREST

The authors declare no competing interests.
