# Supplementary figures and images for: Distinctive incidence patterns of follicular lymphoma in Taiwan: Implications of ethnic differences
Source: Cancer Med. 2019 Feb 21;8(4):1899–907. doi: 10.1002/cam4.2028 (PMC6488204; doi:10.1002/cam4.2028)

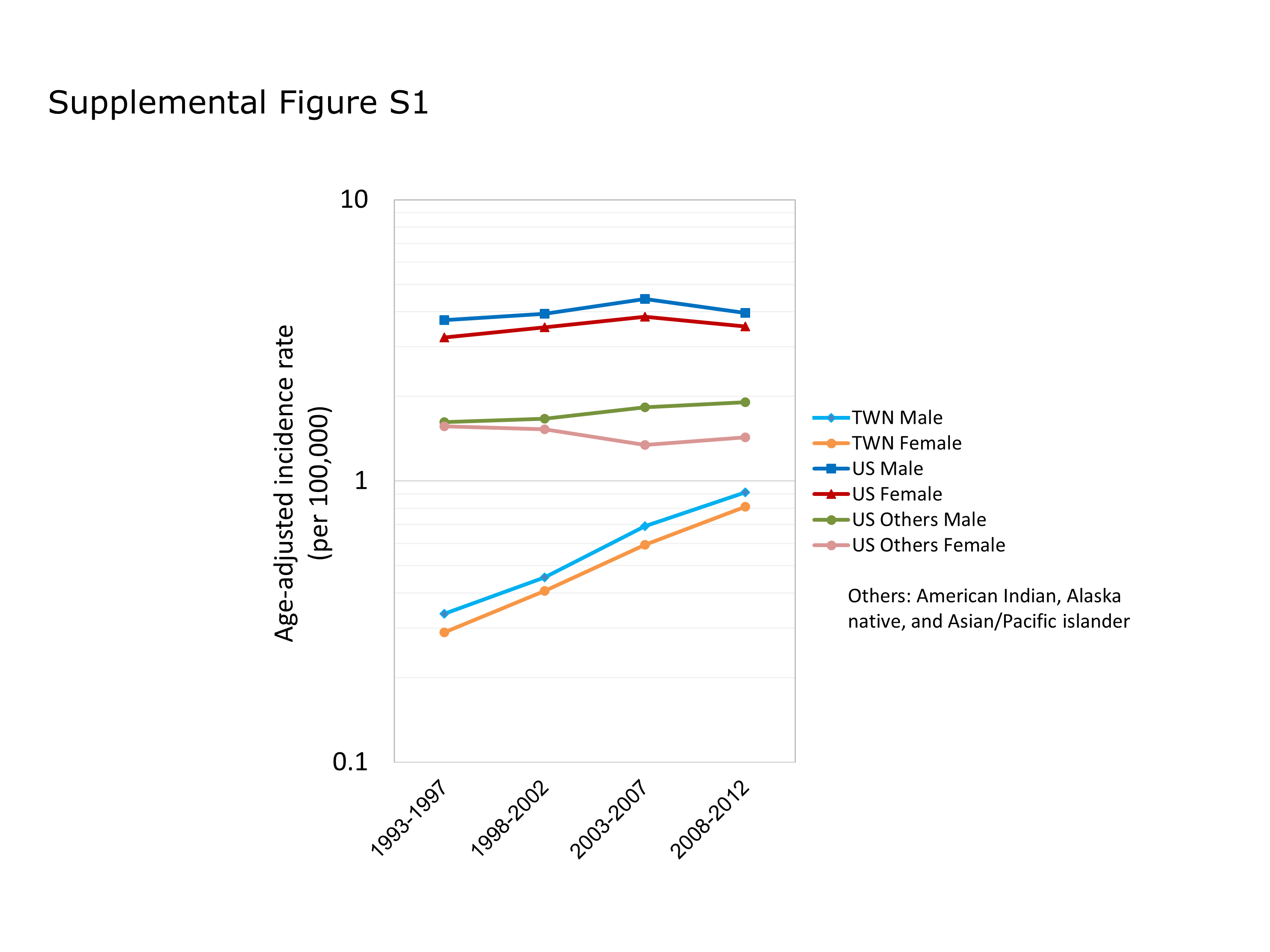

Supplement: Supplementary file 1 [file CAM4-8-1899-s001.tif]

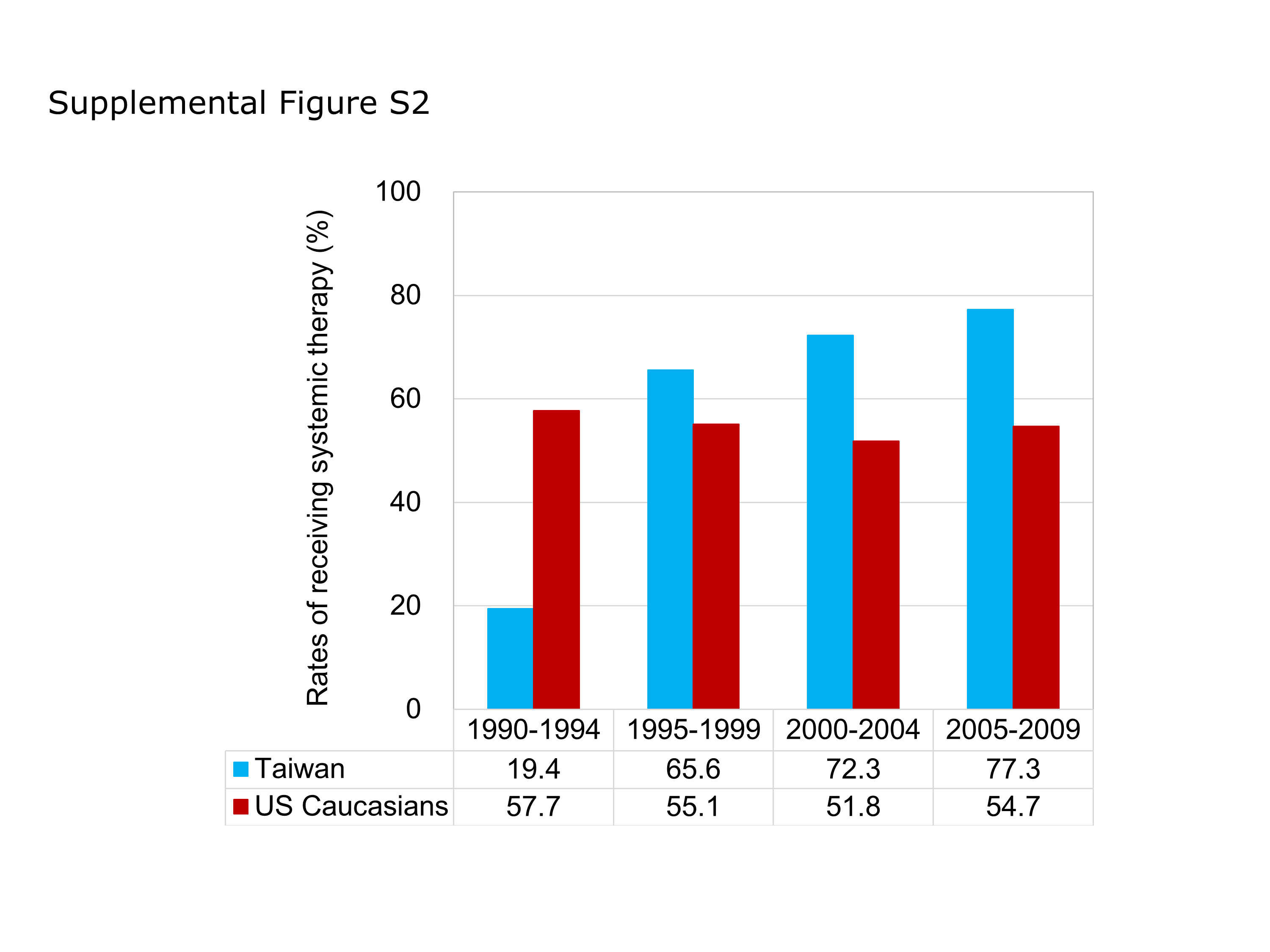

Supplement: Supplementary file 2 [file CAM4-8-1899-s002.tif]
